# The Production and Quality of Different Varieties of Papaya Grown under Greenhouse in Short Cycle in Continental Europe

**DOI:** 10.3390/ijerph16101789

**Published:** 2019-05-20

**Authors:** Mireille N. Honoré, Luis J. Belmonte-Ureña, Asensio Navarro-Velasco, Francisco Camacho-Ferre

**Affiliations:** 1 CIAIMBITAL, Campus de Excelencia Internacional Agroalimentario, Universidad de Almería, Carretera Sacramento s/n, 04120 Almería, Spain; mh052@inlumine.ual.es (M.N.H.); lbelmont@ual.es (L.J.B.-U.); 2 Vitalplant Nursery–Paraje Balsaseca, San Isidro-Níjar, 04117 Almería, Spain; asensio@vitalplant.es

**Keywords:** cultivars of *Carica papaya*, greenhouse, Continental Europe, grafting, yield, total soluble solids

## Abstract

In Europe, papaya consumption is growing due to its nutritional properties. The proximity of consumer markets to Southeast Spain allows fruits to be harvested at a more advanced stage of maturity compared to exporting countries from outside Europe, a timeline which improves the quality of the papaya. Experiments have been carried out to assess the adaptation of papaya to protected cropping systems (under greenhouse) in the region. In this paper, we showed the results obtained in an experiment with five varieties, taking the most cultivated variety as control, which was grafted on its own female rootstock, in addition to another four new varieties that were introduced. Transplanting was made with early sex-identified plants in the nursery. Cultivation was developed in a 446-day cycle, almost 15 months and fruits were always harvested from the soil, due to the height that the plant reached in that period. The best yield parameters and fruit characteristics were obtained from hermaphrodite Intenzza papaya grafted on female papaya rootstock, although there were also other varieties which gave results that made possible its cultivation under this production system.

## 1. Introduction

In 2017, papaya was grown on land of 449,054 ha worldwide. The top five producing countries that year were: India with 5,940,000 t, Brazil with 1,057,101 t, Mexico with 961,768 t, Indonesia with 875,112 t, and the Dominican Republic with 869,306 t. The yield in kilos per unit of area varied greatly depending on the producing countries, from 28.16 kg·m^−2^ in the Dominican Republic to 3.98 kg·m^−2^ in Brazil. Indonesia, Mexico, and India produced between 9.21 and 4.43 kg·m^−2^ [1].

In Europe, papaya consumption is growing due to its nutritional properties. Nowadays, papaya is the third most consumed tropical fruit in Europe. Papaya fruit is a rich source of antioxidant nutrients, B vitamin, potassium, and magnesium, which are elements that prevent and improve digestive system disorders and heart diseases [2].

Cultivation of papaya under greenhouse in Continental Europe has been demonstrated with different experiments carried out in the southern coast of Spain. The varieties assessed in preliminary studies were BH-65, Red lady, Tainung 1, and Siluet [3,4,5]. The proximity of consumer markets to Southeast Spain allows fruits to be harvested at a more advanced stage of maturity compared to exporting countries from outside Europe, a timeline which improves the quality and the taste of the papaya, giving sweeter fruits for consumers. In addition, consumers prefer a papaya with a size from 500 to 800 g (size D to F, Codex STAN 183-1991) [6]. The papaya, like Formosa, is also well valued, but the average weight is more common from 801 to 1800 g (between size G and I, Codex STAN 183-1991) [6]. Varieties with small pieces are generally well valued for retail sale. Conversely, for the industry of fourth range products, varieties with a size higher than size F are also well appreciated. During shelf life, fresh-cut papaya goes through a process of degradation. The flesh color and loss of the texture have an impact on the appearance, which are visual factors of quality for the consumer. On the other hand, the color of the skin gives an idea about its stage of maturity [7]. Furthermore, the contents of soluble solids and the shape of fruits are genetic factors which are associated with quality [6,8]. However, before evaluating all those parameters for the fresh market or for the fourth range products in the industry, researchers of the AGR-200 group of the University of Almeria are concentrated on finding some varieties with a similar higher yield production of the first varieties evaluated in the region and which is generally commercialized in Europe. They are focused on the growth of papaya in greenhouse and want to determine the effect of the plants’ size on the yield crop. In 2016, they conducted an experiment with sex-identified plants in a greenhouse in Almería, which was sowed with different sizes of rootball. The main cultivars which were evaluated and sowed were hybrids of a very small height and low vegetation which allows increasing the planting density in the greenhouse. Varieties like “Sweet Sense”, and “Intenzza” were assessed. On the other hand, the grafting techniques that nurseries in Almería have done for more than 30 years, according to different works made by the AGR-200 research group of the University of Almería, is a technique applicable to the papaya plant following the splice method [9] as it is done in *Solanaceae* species such as tomato and aubergine, therefore, it was decided to conduct this treatment of hermaphrodite papaya plant grafted onto a female plant.

In the present study that was carried out by the AGR-200 group, this time the aims were comparing the agronomic and commercial potentials of different cultivars of papaya, transplanted after their sex-identification and grown in a commercial greenhouse during a short cycle (maximum 18 months) in Southeast Spain and commercialized under European norms. This cycle allows harvesting from the soil, that papaya plants spend only a winter, which is a very unfavourable season for papaya in the region, and in addition to this, it allows using greenhouses with lower structures, which are the greenhouses used by most of the producers in the region.

In high-yielding agriculture of Almería, crops are developed under different greenhouses made of plastic, with different characteristics with respect to areas, heights, and shapes. Plants that have traditionally been grown in these greenhouses are different cultivars of tomato, pepper, aubergine, cucumber, courgette, melon, watermelon, and green bean. Except for tomato and aubergine, the rest of these crops are grown in short cycles, approximately 5–6 months and consequently, two plantations can be done each year. Tomato and aubergine can be grown in short and long cycles. In a long cycle, plantations can last 10–11 months under the greenhouse. In the different papaya experiments that have been carried out in the region, long cycles have been used which leads to keeping papaya plants under cultivation between 22 and 28 months, therefore, the way of harvesting has to be changed because the height that plants reach requires mechanical means to harvest the fruits. This circumstance also requires that cultivation has to be made under high greenhouses (with a minimum distance of 4.5 m to the gutter) and other crops cannot be grown until more than two years have elapsed.

The papaya cultivation is new and reduced in the region and the land. The pathological problems that papaya shows in Southeast Spain are under control.

## 2. Materials and Methods

### 2.1. Experimental Greenhouse and Equipment

**Figure 1:**
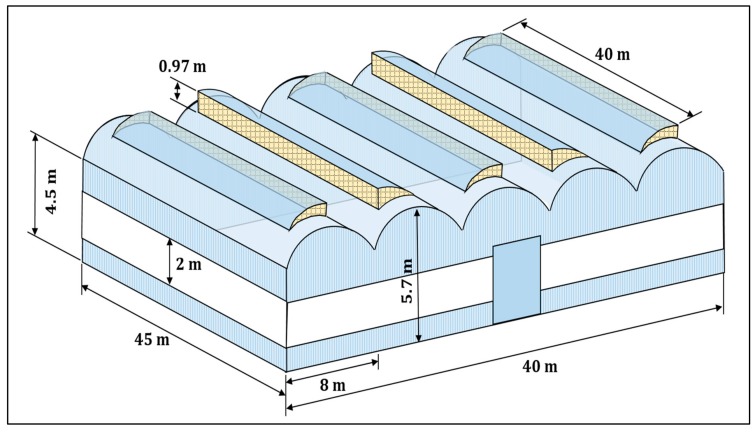
Outline of the experimental trial greenhouse located in the Southeast Spain.

This study was conducted between April 2017 and July 2018, in a 1800 m^2^ multi-tunnel greenhouse in the experimental farm UAL-ANECOOP Center “Catedrático Eduardo Jesús Fernández Rodríguez” of the University of Almería (36°51′ North latitude, 2°17′ West Longitude and altitude of 93 metres above sea level) in Retamar (Almería). The ridges of the greenhouse were east-west oriented. It had 5 modules of the following dimensions: 5.7 m in ridge height, 4.5 m in gutters, 8 m wide, and 45 m long (Figure 1). The crops rows were aligned north to south.

Most of the greenhouse farmers have the Almería-type “raspa y amagado” greenhouse, the most common in the area, with a maximum height of 4 m [10]. To reproduce the dimension conditions in this particular type of greenhouse for the present study, the monitoring of the crop was carried out only during a short production cycle in a multi-tunnel type greenhouse.

The covering of the greenhouse was made of a three-layer thermal plastic of 200 microns thick, low density polyethylene film (LDPE). The greenhouse had zenithal windows vents in the five modules, which worked with engines and a rack and pinion mechanism, whose opening was controlled by temperature and wind sensors. In this part of the Andalusian region from June to September, the prevailing winds blow from the east to west, and it is a hot wind. To avoid the decrease of the relative humidity inside the greenhouse, white plastic mulch film was placed on the soil, in alternate lines, forming small reservoirs which were filled with water to produce evaporation. In winter, this much film was not filled with water and the alternate lines were mulched with black plastic film. This method avoided some fungal diseases as covering the whole soil surface decreased relative humidity. During the winter period, the greenhouse was maintained with the side walls closed. In mid-May, the side walls were opened.

### 2.2. Climatic Conditions

**Figure 2:**
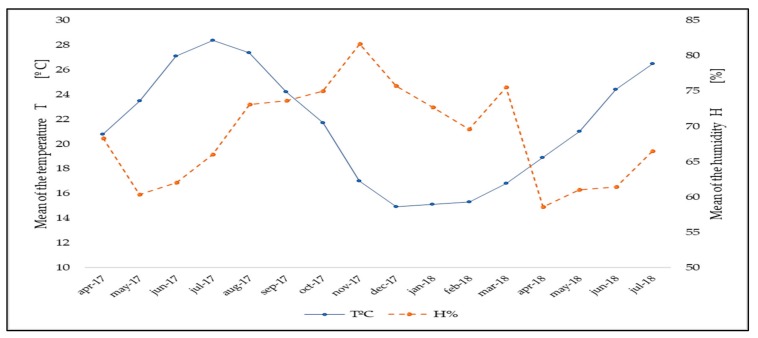
Average of the temperature T (-): and humidity H (**---**) inside the greenhouse during the experiment.

The temperature and humidity were measured at 4 m above the ground with the Elektronik E+E210 sensor, with a temperature range of (−20 °C to +80 °C) and a humidity range of (0%−100%). With the ventilation conditions stated as previously, the temperature and humidity were the following. During the experiment, the coldest month was December 2017 with the average minimum temperature of 14.9 °C and the coldest day being 3.8 °C. July 2017 was the hottest month with the average maximum temperature of 28.4 °C and the hottest day being 42.3 °C. With respect to relative humidity, November 2017 was the month with the highest average humidity of 81.7% and recorded an absolute maximum of 98.4%. The lowest average humidity was 60.3% in May 2017 and in February 2018 was recorded the minimum absolute humidity of 18.9% (Figure 2).

### 2.3. Experimental Procedures

The cultivation was carried out in a soil protected with sand, which is typical in this region, as described by Camacho and Fernández the most used-in greenhouses of Almería [11,12]. A total of 10 kg·m^−2^ of fresh manure were added to the soil. Therefore, the nutrients and the organic matter were adequate within the range of fertile soils. Tomato, pepper, courgette, and cucumber were grown in this soil for 13 years.

A drip irrigation system was used with flow rate emitters of 3 L·h^−1^ and a density of 1.6 emitter/m^−2^. Fertilization and irrigation were applied simultaneously with the drip irrigation. The inline lateral pipes were north-south oriented, as the plants are. From April to October 2017, the irrigation control was made with tensiometers. The range of control was 20–10 cbar, and from November to 18th June of 2018, the range was 35–18 cbar.

### 2.4. Fertigation

The soil had a clay loam texture. The soil pH was 7.80, and organic matter content was 0.45%. The electrical conductivity (EC) values, in the saturation extract, was 4.65 dS·m^−1^.

The water used had an EC of 1.4 dS·m^−1^ and a pH of 7.13. The fertigation was applied with the drip irrigation to obtain the following maximum concentrations in the solution: NO_3_^−^: 16 mmol·L^−1^; H_2_PO_4_^−^: 1.4 mmol·L^−1^; SO_4_^2−^: 1.7 mmol·L^–1^; HCO_3_^−^: 0.5 mmol·L^−1^; K^+^: 8 mmol·L^−1^; Ca^2+^: 6 mmol·L^−1^; and Mg^2+^: 2 mmol·L^−1^. A fertilizer called “UniquaTrop^®^”, made by the company Megasa was specifically made for this purpose with the following composition: NPK+Ca+Mg: 2.9–1.3–5.7 (2.2–0.5).

Seven days after transplanting (dat), the addition of the fertilizer began. The EC range (+0.5 a +2.0) dS·m^−1^ of irrigation water increased progressively and it never exceed 0.5 dS·m^−1^ in the increases made. A mix of microelements were added to this fertilizer with 7.5% of Fe, 2.5% of Mn, 0.15% of Cu, 0.1% of Zn, 1.25% of B, and 0.25% of Mo. The rate of this mix was 20 g·m^−3^ of irrigation water used.

### 2.5. Field Layout and Plant Material

Four papaya varieties were planted to assess the potential yield of each variety and to compare them with the most used variety under greenhouse in Southeast Spain, which is the Intenzza variety. Another treatment was also made with this Intenzza variety; it was grafted on a female rootstock of the same variety. All the varieties were early sex-identified when they were in a cotyledon state, 40 days after sowing (das) in Vitalplant nursery, in such a way that the transplant was made with one plant per pot. The plants were prepared in the nursery with an approximate height of 60 cm, in a rootball pot of 2 L. The treatments made were:

T0: Intenzza.
T1: Hermaphrodite Intenzza onto female Intenzza.
T2: Sweet Sense.
T3: Vitale.
T4: Caballero.
T5: Alicia.

The total area of the experiment was 588.84 m^2^. Three replications for each treatment were made. Each elemental plot was 32.7 m^2^. The elemental plots were distributed at randomized blocks. The experiment was carried out in the north side of the greenhouse, and a plot of 266.6 m^2^ was used as edge effect plants. The south plot of the greenhouse (both separated by a central corridor) of 855 m^2^ was also planted with papaya. The area of the corridor was 90 m^2^. The planting density was 2700 plants/ha. The distribution of plants was made in paired and staggered rows, with 2.20 m between paired rows, 1.00 m between lines, and 2.00 m between plants.

Since 2014, in the southeast of Spain, different experiments have been carried out in papaya cultivation to find a variety which gives fruits a weight between 600 and 1500 g, and that plants have a good behavior with respect to soil conditions, water, and climate. The characteristics of the cultivars used according to the companies that produce the seeds are described in Table 1 [6,13,14,15,16].

### 2.6. Indices Maturity Stage for Harvesting

The fruit harvest was carried out every week, from 160 dat to 446 dat (from October 2017 to July 2018). The fruits were harvested in the maturity stages 2 and 3 described by Santamaria [7]. The maturity stage 2 corresponds with a visual aspect of green fruit but with a light-yellow coloring between 25%–33%. The maturity stage 3 corresponds with yellow coloring fruit between 34%–40%. From spring the harvest was only carried out at maturity stage 2. The harvest was carried out from one day to four days per week; it increased as temperatures were higher.

### 2.7. Data Collection and Statistical Analysis

The parameters assessed were: Yield, measured by kg·m^−2^, fruit weight (kg), number of fruits per plant, total soluble solids (TSS) measured by ° Brix. Fruits that were at a maturity stage higher than 3, those with deformations, or those damaged fruits were discarded. The fruits which had to be analyzed to know the total solubles solids (° Brix), were weighted with a Classic ML6001E (Mettler Toledo, L’Hospitalet de Llobregat, Spain) precision scales, with a maximum capacity of 6200 g and ±0.1 g sensitivity. The result of TSS in fruits was obtained with the average ° Brix and the maximum ° Brix.

Every four weeks, the TSS of fruits was measured. From each of the treatments, a fruit was chosen at random. The data was obtained between weeks 10 and 25 in 2018. A digital Pal-1 (Atago Co., LTD., Tokio, Japan) refractometer that had a measuring range from 0–53 ° Brix and ±0.2 sensitiviy was used. The process was repeated three times per each fruit, and every time a different piece was used. The refractometer was calibrated with distilled water.

This fruit was peeled and cut lengthwise (weight higher than 15% of the total weight of harvested fruit). This piece of pulp was mashed with a kitchen press, and the juice extracted from the mashed pulp (8–10 g) was measured to know the ° Brix.

After the first measurement, the fruit chosen at random for each treatment was kept in a cooling chamber at 15 °C. The same process was repeated in the same way and three new measurements of TSS were obtained 3, 7, and 10 days after the first measuremet. The results analyzed were obtained from five harvests. The measurements were taken at room temperature from 19 °C in March to 32 °C in June.

Additionally, morphological data about the plant was gathered, specifically: Height from the peduncle of the first harvested fruit to soil, which was taken in week 40 in 2017. On this same date, the perimeter of the stem was measured, calculating the diameter of the same, and this data was taken when 10 cm from the soil. The number of nodes from the soil to the first fruit was measured in weeks 40 and 43 of 2017 and weeks 9, 10, 12, 14, 15, and 16 in 2018. The morphological data was taken using a measuring tape with a total length of 5 m and was graduated in mm.

The data was subjected to variance analysis (ANOVA), considering a significant data if *p* ≤ 0.05. The average values were compared using the Fishers Least Significant Difference (LSD), using the computer software STATGRAPHICS^®^ Centurion XVIII version 18.1.06 (64 bits) (Statpoint Technologies, Warrenton, United States of America, Inc. 1982–2018).

## 3. Results

### 3.1. Foliar Analysis

In October 2017 a foliar analysis was made and the parameters that were measured (N, P, K, Ca, Mg, Na, Fe, Mn, B, Cu, Zn, Mo), showed normal results in the petiole of the papaya leaf. The analysis was made in that tissue because the content of minerals is more stable than in the leaf blade. The sufficiency range of the nutrients found in the petiole of the papaya leaf are taken from Jones et al. (1991), according to the data shown by Osuna Enciso [17]. The results for each element analyzed were the following: N (1.26 %), P (0.21%), K (3.43%), Ca (1.09%), Mg (0.48%), Fe (28.5 ppm), Mn (23 ppm), B (29 ppm), Cu (5 ppm), and Zn (17 ppm). The range described by Osuna Enciso was: N (1%–2.5%), P (0.2%–0.4%), K (3.3%–5.5%), Ca (1%–3%), Mg (0.4%–1.2%), Fe (25–100 ppm), Mn (20–150 ppm), B (20–30 ppm), Cu (4–10 ppm), and Zn (15–40 ppm).

### 3.2. Crop Protection

In the experiment, there were problems with *Tetranichus urticae* attacks that were controlled with *Phytoseiulus persimilis* and *Amblyseiuscalifornicus* releases; *Stethorus punctillum* also appeared sporadically, which is a big predator of *Tetranichus* sp. Small attacks of *Aphis gossypii* were controlled with preventive *Lysiflebus testaceipes* and *Aphidius colemani* releases on cereal banker plants, which were infected with the specific aphid, *Rhopalosiphum padi*. In winter, small attacks of *Botrytis cinerea* appeared on the injuries in the stem, which caused the fall or removal of the petioles, and was controlled with Samurai^®^–Nutricrop paste (a product made from natural clays that isolates the internal tissues of the plant from the outside environment). At the end of winter, beginning of spring, *Oidium caricae* attacks appeared which were controlled by applying powdered sulphur which we alternated with products such as Ospo V^®^- Agrotecnología (a product made from vegetal extracts, flavonoids, alkaloids, phenols, macro and microelements, polysaccharides, and microorganism extracts) and Lareki greens^®^, Biofungitek, (a product made from potassium carbonate and vegetal extracts).

### 3.3. Total Yield

**Figure 3:**
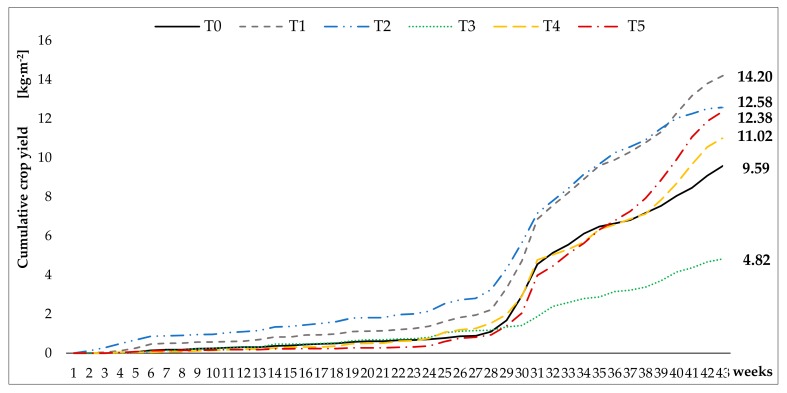
Cumulative crop yield of the five cultivars on 43 weeks in (kg·m^−2^), T0 (█): Intenzza; T1 (█): Grafted Intenzza; T2 (█): Sweet sense; T3 (█): Vitale; T4 (█): Caballero; T5 (█): Alicia planted under greenhouse.

The harvest was carried out for 43 weeks. There were significant differences between treatments, T1 (Grafted Intenzza) was the treatment from which more yield was obtained, with 14.20 kg·m^−2^. T2 (Sweet Sense) and T5 (Alicia) treatments form another statistical group that obtained 12.58 and 12.38 kg·m^−2^ respectively, the rest of the treatments were slightly lower in yield (Table 2). From week 4 of harvest, T1 was the treatment which gave more yield, and T3 treatment kept the harvest pace until week 25, the remaining treatments obtained always lower yields, although the significant increase of T4 and T5 from week 27 to 30 must be highlighted (Figure 3).

### 3.4. Average Fruit Weight (AFW)

**Figure 4:**
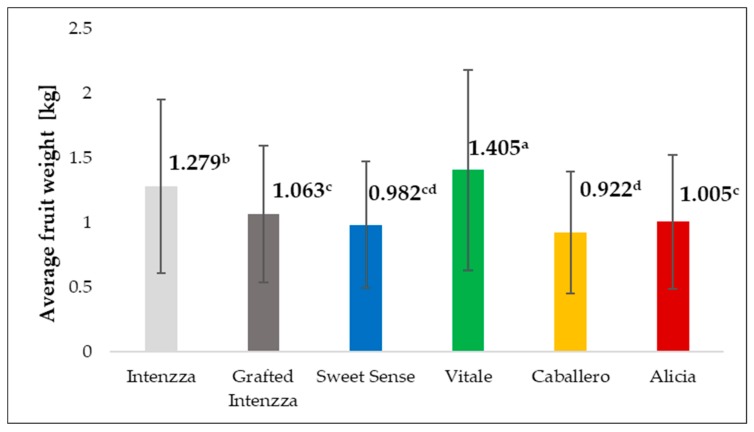
Average fruit weight of the five cultivars of papaya in (kg). T0 (█): Intenzza; T1 (█): Grafted Intenzza; T2 (█): Sweet sense; T3 (█): Vitale; T4 (█): Caballero; T5 (█): Alicia, planted under greenhouse. Different letters mean significant differences, at *p*-value < 0.05.

Throughout the harvest, significant differences were observed between the fruits coming from the different treatments. The Vitale fruits had the highest weight, with a value of 1.405 kg, followed by T0 (Intenzza) fruits with 1.279 kg (Table 2), both values corresponded with size H (1104–1500 g) according to Codex 183–93, the rest of the fruits coming from the remaining treatments were classified as size G (801–1100 g) (Figure 4).

In Europe, the major papaya importers market papaya per units and not by weight (retail sale), therefore, the “average fruit weight” parameter has importance with respect to fruit marketing, distribution, and packaging. Fruit weights between 600 to 1000 g have an acceptable demand by European consumers and they can be sold in many countries.

### 3.5. Number of Fruits Per Plant

**Figure 5:**
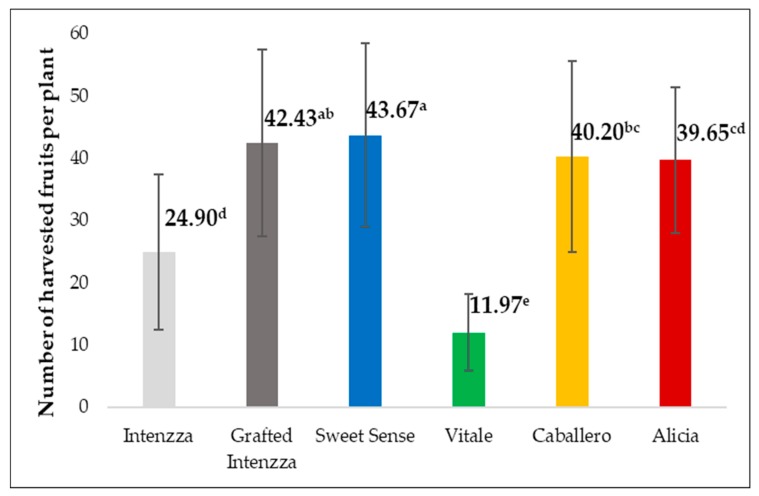
Harvested fruits per plant of the five cultivars of papaya cultivated under greenhouse. Different letters mean significant differences, at *p*-value < 0.05.

There were significant differences in this parameter for all the treatments (Table 2). Sweet Sense and Intenzza grafted on its own female rootstock were the plants with the highest number of fruits (Figure 5).

### 3.6. Total Soluble Solids

**Figure 6:**
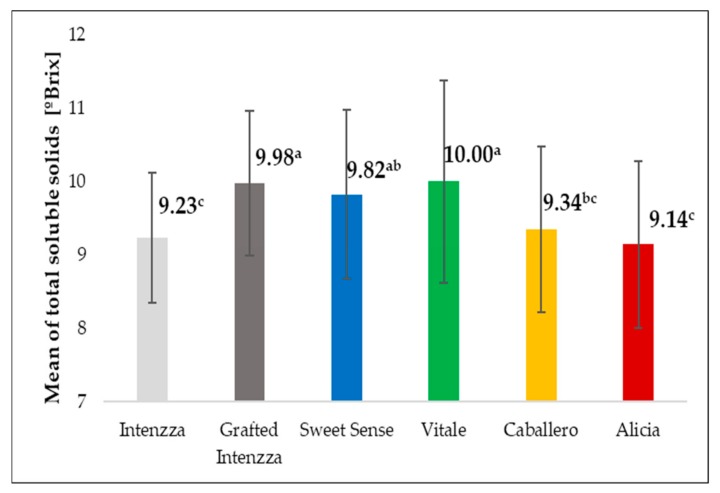
Average of the total soluble solids (TSS) measured of fruits of papaya cultivated under greenhouse. Different letters mean significant differences, at *p*-value < 0.05.

The analysis of total soluble solids was made for the ° Brix average, and for the maximum and minimum ° Brix in fruits (Figure 6). There were significant differences between treatments in the three measurements. T3 and T1 (Vitale and grafted Intenzza) form the group of fruits with the highest ° Brix, and the fruits coming from Alicia and Intenzza (T5 and T0) form the group of fruits with the lowest TSS. The same situation happened when the average of the maximum and minimum ° Brix was analyzed. The average minimum values obtained varied from 9.59 to 8.76 ° Brix. The absolute maximum values had values between 9.14 and 10.00 ° Brix.

An increase in the TSS can be observed in the measurement taken 10 days after the harvest (10 days after the first measurement (D + 10)). The value reached 10.33% if the absolute maximum values are considered (Table 2). All the measurements for this parameter taken after the harvest was carried out resulted in a value higher than the previous ones in the same fruit.

### 3.7. Height from the Soil and Node in which the First Flower Appears

**Figure 7:**
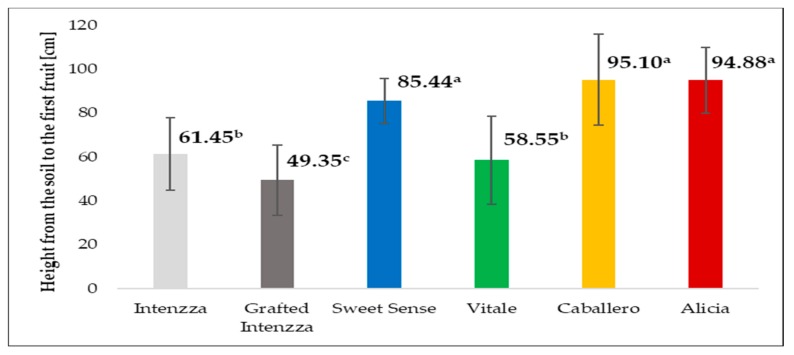
Height from the soil to the first fruit measured for five cultivars of papaya planted under greenhouse. Different letters mean significant differences, at *p*-value < 0.05.

There were significant differences between treatments, with respect to the height between the soil and where the first fruit was harvested, T4 and T5 (Caballero and Alicia) are the treatments where the first highest fruit was harvested, followed by T2 (Figure 7).

**Figure 8:**
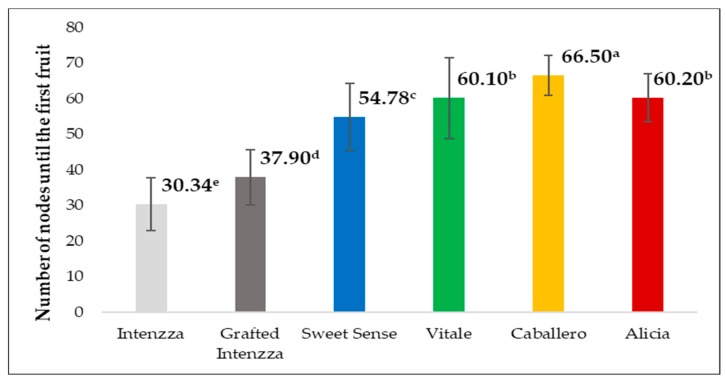
Number of nodes until the first fruit for the five cultivars of papaya planted under greenhouse. Different letters mean significant differences, at *p*-value < 0.05.

With respect to the first node where the harvest was carried out, there were also significant differences and it also coincided with T4 (Caballero) in which the highest number of nodes appeared (Figure 8). The relation between both parameters gave the distance between nodes, which varied from 1.30 cm for Intenzza grafted onto its female rootstock to 2.02 cm of non-grafted Intenzza (T0).

### 3.8. Perimeter of the Stem

**Figure 9:**
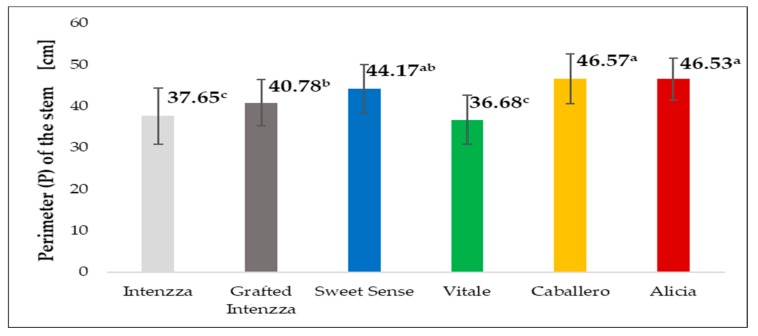
Perimeter (P) of the stem for the five cultivars of papaya planted under greenhouse. Different letters mean significant differences, at *p*-value < 0.05.

There were significant differences between treatments (Table 2). T4 and T5 (Caballero and Alicia) had the highest longitudes of the perimeter, 46.57 and 46.53 cm respectively. The lowest longitude of perimeter was obtained with Vitale and Intenzza with 36.68 and 37.65 cm respectively (Figure 9). These perimeters gave a diameter of the stem between 11.68 and 14.82 cm for the thinnest stem (Vitale) and for the thickest (Caballero) respectively, as it can be appreciated there is a 3.14 cm gap.

## 4. Discussion

Our yield results were higher than those obtained by Guzmán [18] which varied between 4–7 kg·m^−2^ and Jiménez [19] who obtained 8.9 kg·m^−2^, both in Costa Rica and the last one in a three-year cycle. In Mexico, Escamilla et al. [20] conducted experiments to study the effects of organic, mineral, and foliar fertilization on the development and yield of *cv* Maradol papaya and obtained 2.8 kg·m^−2^. In India, Singh et al. [21] conducted experiments to assess the effects of micronutrients on growth, yield, and quality of papaya, and reported yields between 6.5–9.3 kg·m^−2^. In other experiments in India, Bhalerao et al. [22] obtained between 5.18–8.07 kg·m^−2^ in an experiment that assessed the effects of different micronutrients on yield and quality of papaya. In all the cases that have been compared, the plant cycles in field were longer than those of our experiment, which makes possible papaya cultivation in Southeast Spain in Europe, giving higher yields because the cycle was reduced until 30% compared with the rest of the plantation systems. In our treatments, an improvement can be appreciated when using hermaphrodite plant grafted onto female plant compared with non-grafted plants. The reason is that female and hermaphrodite plants are older than non-grafted plants, although they were planted the same day, the seed of grafted plants was sowed 35 days before, with the purpose that growing at the transplanting stage was similar.

With respect to fruit weight, the data obtained are consistent with the data obtained by Pérez [23], in an experiment conducted with 10 different varieties, although the production system was not the same. All the treatments gave fruits between 600 and 1200 g, a size demanded by the market which is accessible from Almería.

It can be observed in the different varieties, the significance of the genetic power on fruit size and weight. The weight obtained in the fruits coming from the different treatments has an acceptable demand in the European market, and they can be sold in many countries.

The number of fruits obtained in each of the varieties can be considered as high, hence its repercussion on yield for short cycle. Comparison with other varieties, as used in other experiments, would provide nothing to this parameter.

In soluble solids, our values were similar to those obtained by Pérez [23] for the Intenzza variety grown in the Canary Islands, although, in this case, two peaks of 12 ° Brix were observed. The values are also similar to the results obtained by Santamaría [24] in Costa Rica. The company that produces the Intenzza variety [14] claims that the ° Brix are between 10–13. In the case of Sweet Sense [15], the figures obtained are between 11 and 14 º Brix.

The increase of ° Brix of the papaya after harvest have been reported by other authors, as Siriamornpun et al. [25] observed this phenomenon in papayas of different cultivars (KhakDam, Hawaii, and Holland), which were left to be ripened in 24 and 48 h at room temperature, compared with other group of green papayas. The ripe papaya they analyzed was fully yellow. They attribute the increase of TSS to the fact that fruit during the ripening process have more intense cellular respiration processes due to hydrolisis of starch to glucose and fructose by amylase action, described by Eskin et al. [26]. In our experiment, fruits were kept cool at 15 °C (except for when measurements were taken at room temperature). The data obtained in the experiment permitted the sale of papaya fruits in different European countries. Once again, the position of this parameter of hermaphrodite Intenzza grafted onto female Intenzza must be highlighted.

The results obtained with respect to the height of the first fruit, are consistent with those reported by Alonso et al. [27] for two varieties belonging to the germplasm bank located in the Scientific-Technological Unit (UCTB) in Jagüey Grande, Matanzas (Cuba) and those obtained by Pérez [23] for Intenzza and those obtained by Escamilla et al. [20] in Mexico for the Maradol variety.

This perimeter is slightly equal to the perimeter obtained by Alonso et al. [27] in Cuba, although the authors did not state when the measurement was taken with respect to transplanting. The diameters obtained according to the plant short cycle were enough to support the load of the fruits and the plant was neither bent nor had fallen over at any time.

If we analyze all the assessed parameters, we should consider grafting hermaphrodite plants onto female plants due to the benefit of early sex-identification plants, in addition to plants being transplanted with a higher development.

## 5. Conclusions

The most productive treatment corresponded with the hermaphrodite Intenzza variety grafted onto the female Intenzza rootstock. In all the treatments, the fruit weight reached the marked weight for our objective, which makes possible the selling of these fruits in European markets. Sweet Sense and hermaphrodite Intenzza grafted onto female Intenzza rootstock are the varieties which gave a higher number of fruits. When harvest was carried out, all the treatments had between 9–10 ° Brix, then the climacteric effect on the fruits was an increase by 10.33%, 10 days after the harvest and kept at 15 °C. Hermaphrodite Intenzza grafted on female Intenzza rootstock was the plant where the first fruit was harvested at the lowest distance from the soil, and its diameter of the stem was within the lowest thickness group. In all the treatments assessed, hermaphrodite Intenzza grafted onto female Intenzza obtained the best parameters to be an alternative to be considered with respect to other varieties.

## Figures and Tables

**Table 1:** Characteristics of the five cultivars of papaya, planted in this experiment, under greenhouse.

Variety (Seller) [13,14,15,16]	Fruit Weight (g)	Size FAO * [6]	Main Characteristics	Sweetness Total Soluble Solids (° Brix)
Intenzza (Semillas del Caribe)	1500–2200	I, J	Developed for medium size or Formosa type papaya market. Its epidermis is orange-yellow when it is ripe, and its interior is red.	10–13
Sweet Sense (Semillas del Caribe)	1200–1800	H, I	When it is ready to be consumed, on its ideal ripening state, its epidermis is yellow, and its interior is salmon. It is classified as “baby” type.	11–14
Vitale (Vitalplant Nursery)	>1000	G, H, I, J	It is a plant with medium vigor, very open.	Without information from the seller
Caballero (CapGenSeeds)	650–900	E, F, G	It is not a very big plant and it has few leaves. It is ideally grown under greenhouse. Its fruits are very sweet.	14–15
Alicia (CapGenSeeds)	750–1100	F, G	It is not a very high plant; therefore, it is ideal to grow under greenhouse. The internodes of the plant are very short. Alicia tolerates low temperatures. Fruit harvest can be carried out in an advance ripening stage.	Without information from the seller

* Food and Agriculture Organization of the United Nations (FAO).

**Table 2:** Results obtained in papaya grown under greenhouse: Yield (kg·m^−2^), Average fruit weight (AFW), number of harvested fruits, height from the soil to the first fruit, perimeter of the stem, number of nodes until the first fruit, Total soluble solids (TSS) (° Brix).

Treatments	Intenzza	Grafted Intenzza	Sweet Sense	Vitale	Caballero	Alicia	*p*-Value
	T0	T1	T2	T3	T4	T5	
Yield (kg·m^−2^)	9.59 ^c^	14.20 ^a^	12.58 ^ab^	4.82 ^d^	11.02 ^bc^	12.38 ^ab^	0.00
*SIZE (FAO)* AFW (kg)	*H* 1.279 ^b^	*G* 1.063 ^c^	*G* 0.982 ^cd^	*H* 1.405 ^a^	*G* 0.922 ^d^	*G* 1.005 ^c^	0.00
Number of harvested fruits per plant	24.90 ^d^	42.43 ^ab^	43.67 ^a^	11.97 ^e^	40.20 ^bc^	39.65 ^cd^	0.00
TSS (° Brix)	9.23 ^c^	9.98 ^a^	9.82 ^ab^	10.00 ^a^	9.34 ^bc^	9.14 ^c^	0.00
MAX TSS (° Brix)	9.72 ^bc^	10.45 ^a^	10.19 ^ab^	10.36 ^a^	9.72 ^bc^	9.50 ^c^	0.00
MIN TSS (° Brix)	8.76 ^c^	9.51 ^ab^	9.46 ^ab^	9.59 ^a^	9.01 ^bc^	8.77 ^c^	0.00
Increase of TSS (° Brix) 1st to 4th measurement Average values	10.1 ^a^	8.6 ^a^	10.3 ^a^	9.2 ^a^	9.1 ^a^	9.0 ^a^	0.99
Increase of TSS (° Brix) 1st to 4th measurement Maximum values	11.8 ^a^	11.9 ^a^	8.8 ^a^	9.7 ^a^	9.5 ^a^	10.3 ^a^	0.99
Increase of TSS (° Brix) 1st to 4th measurement Minimum values	8.1 ^a^	8.7 ^a^	11.1 ^a^	7.2 ^a^	8.6 ^a^	8.3 ^a^	0.99
Height from the soil to the first fruit (cm)	61.45 ^b^	49.35 ^c^	85.44 ^a^	58.55 ^b^	95.1 ^a^	94.88 ^a^	0.00
Perimeter of the stem (cm)	37.65 ^c^	40.78 ^b^	44.17 ^ab^	36.68 ^c^	46.57 ^a^	46.53 ^a^	0.00
Diameter of the stem	11.98 ^a^	12.98 ^b^	14.06 ^bc^	11.68 ^a^	14.82 ^c^	14.81 ^c^	0.00
Number of nodes until the first fruit	30.34 ^e^	37.90 ^d^	54.78 ^c^	60.10 ^b^	66.50 ^a^	60.20 ^b^	0.00

Different letters mean significant differences, at *p*-value < 0.05.

## References

[1] FAO (2017). Crop Data.

[2] Karunamoorthi K., Hyung-Min K., Jegajeevanram K., Xavier J., Vijayalakshmi J. (2014). Papaya: A gifted nutraceutical plant—A critical review of recent human health research. Tang Humanit. Med..

[3] Hueso J.J., Schmildt R., Schmildt O., Cuevas J. (2017). Comparación de los Sistemas Productivos de la Papaya en España y Brasil. Innovagri. https://www.innovagri.es/investigacion-desarrollo-inovacion/comparacion-de-los-sistemas-productivos-de-la-papaya-en-espana-y-brasil.html.

[4] Hueso J.J., Salinas I., Pinillos V., Cuevas J. (2017). El Cultivo de la Papaya en el Sureste de España. Horticultura—Interempresas. http://www.interempresas.net/Horticola/Articulos/196398-El-cultivo-de-la-papaya-en-el-Sureste-de-Espana.html.

[5] Salinas I., Hueso J.J., Cuevas J. (2019). Fruit growth model, thermal requirements and fruit size determinants in papaya cultivars grown under subtropical conditions. Sci. Hortic..

[6] FAO (2011). Norma para la Papaya. CODEX STAN 183-1993. Food and Agriculture Organization of the United Nations (FAO). CODEX Alimentarius. International Food Standars. http://www.fao.org/fao-who-codexalimentarius/sh-proxy/es/?lnk=1&url=https%253A%252F%252Fworkspace.fao.org%252Fsites%252Fcodex%252FStandards%252FCODEX%2BSTAN%2B183-1993%252FCXS_183s.pdf.

[7] Santamaría-Basulto F., Díaz-Plaza R., Sauri-Duch E., Espadas y Gil F., Santamaría-Fernández J.M., Larqué-Saavedra A. (2009). Quality characteristics in maradol papaya fruits at the comsumption ripeness stage. Agric. Téc. Méx..

[8] Nantawan U., Kanchana-udomkan C., Drew R., Ford R. (2018). Identification of genes related to sugar content in *Carica papaya* L.: Differential expression and candidate marker development. Acta Hortic..

[9] Miguel Gómez A. (2011). El injerto de plantas de tomate. Serie documentos de la editorial THM. https://issuu.com/horticulturaposcosecha/docs/el_injerto_de_plantas_de_tomate?e=8490508/66927659.

[10] Valera D.L., Belmonte L.J., Molina-Aiz F.D., López A., Camacho F. (2017). The greenhouses of Almería, Spain: Technological analysis and profitability. Acta Hortic..

[11] Valera-Martínez D.L., Belmonte-Ureña L.J., Molina-Aiz F.D., López-Martínez A. (2016). Greenhouseagriculture in Almería. A Comprehensive Techno-Economicanalysis.

[12] Camacho-Ferre F., Fernández-Rodríguez E.J. (2000). El Cultivo de Sandía Apirena Injertada, bajo Invernadero, en el Litoral Mediterráneo Español.

[13] Semillas del Caribe (2015). Ficha técnica de la semilla de papaya Intenzza. http://www.semillasdelcaribe.com.mx/wp-content/uploads/2015/10/FichaTecnicaIntenzza.pdf.

[14] Semillas del Caribe (2015). Ficha técnica de la semilla de papaya Sweet Sense. http://www.semillasdelcaribe.com.mx/wp-content/uploads/2015/10/FichaTecnicaSweetSense.pdf.

[15] (2016). Capgenseeds Datos Técnicos sobre la papaya Caballero. http://www.capgenseeds.com/es/venta-semillas/comprar-semillas-papayas/papayacaballero.

[16] Capgenseeds (2016). Datos Técnicos sobre la Papaya Alicia. http://www.capgenseeds.com/es/venta-semillas/comprar-semillas-papayas/papaya-alicia.

[17] Osuna-Enciso T., Escobar-Álvarez J.L., Nolasco-González Y., Muy-Rangel M.D., Rubio-Carrasco W., Contreras-Martínez R., Becerra-Leor E.N., Obando-Cruz M.E. (2015). Proyecto 60135: El manejo integral del cultivo de Papaya en México, un Acercamiento innovador. Estado nutricional y calidad del fruto de papaya en Veracruz, Oaxaca y Colima, México. http://sistemanodalsinaloa.gob.mx/archivoscomprobatorios/_16_informetecnicoconsultorias/7615.pdf.

[18] Guzmán G. (1998). Guía para el cultivo de la papaya (Carica papaya L.).

[19] Jiménez J.A. (2002). Manual práctico para el cultivo de la papaya Hawaiana.

[20] Escamilla J.L., Saucedo C., Martínez M.T., Martínez A., Sánchez P., Soto M. (2003). Organic, Mineral and Foliar Fertilization on Development and Production of Papaya cv.Maradol. Terra Latinoam..

[21] Singh D.K., Ghosh S.K., Paul P.K., Suresh C.P. (2010). Effect of Different Micronutrients on Growth, Yield and Quality of Papaya (*Carica papaya* L.) cv. Ranchi. Acta Hortic..

[22] Bhalerao P.P., Patel B.N., Patil S.J., Gaikwad S.S. (2014). Effect of foliar application of Ca, Zn, Fe and B on growth, yield and quality of papaya (*Carica papaya*) cv. Taiwan Red Lady. Curr. Hortic..

[23] Pérez-Hernández E. (2016). Ensayo de variedades de papaya 2013–2015.

[24] Santamaría F., Mirafuentes F., Zavala M.J., Vázquez E. (2015). Fruitquality of red papaya genotypescultivated in yucatan, Mexico. Agronomíacostarricense.

[25] Sirithon S., Niwat K. (2017). Quality, bioactive compounds and antioxidant capacity of selected climacteric fruits with relation to their maturity. Sci. Hortic..

[26] Eskin N.A.M., Hoehn E., Shahidi F., Eskin N.A.M., Shahidi F. (2013). Fruits and vegetables. Biochemistry of Foods.

[27] Alonso M., Tornet Y., Ramos R., Farrés E., Aranguren M., Rodríguez D. (2008). Characterization and evaluation of two papaya hybrids in Cuba. Agric. Téc. Méx..

